# Supplementary figures and images for: Cbln1 regulates axon growth and guidance in multiple neural regions
Source: PLoS Biol. 2022 Nov 17;20(11):e3001853. doi: 10.1371/journal.pbio.3001853 (PMC9671368; doi:10.1371/journal.pbio.3001853)

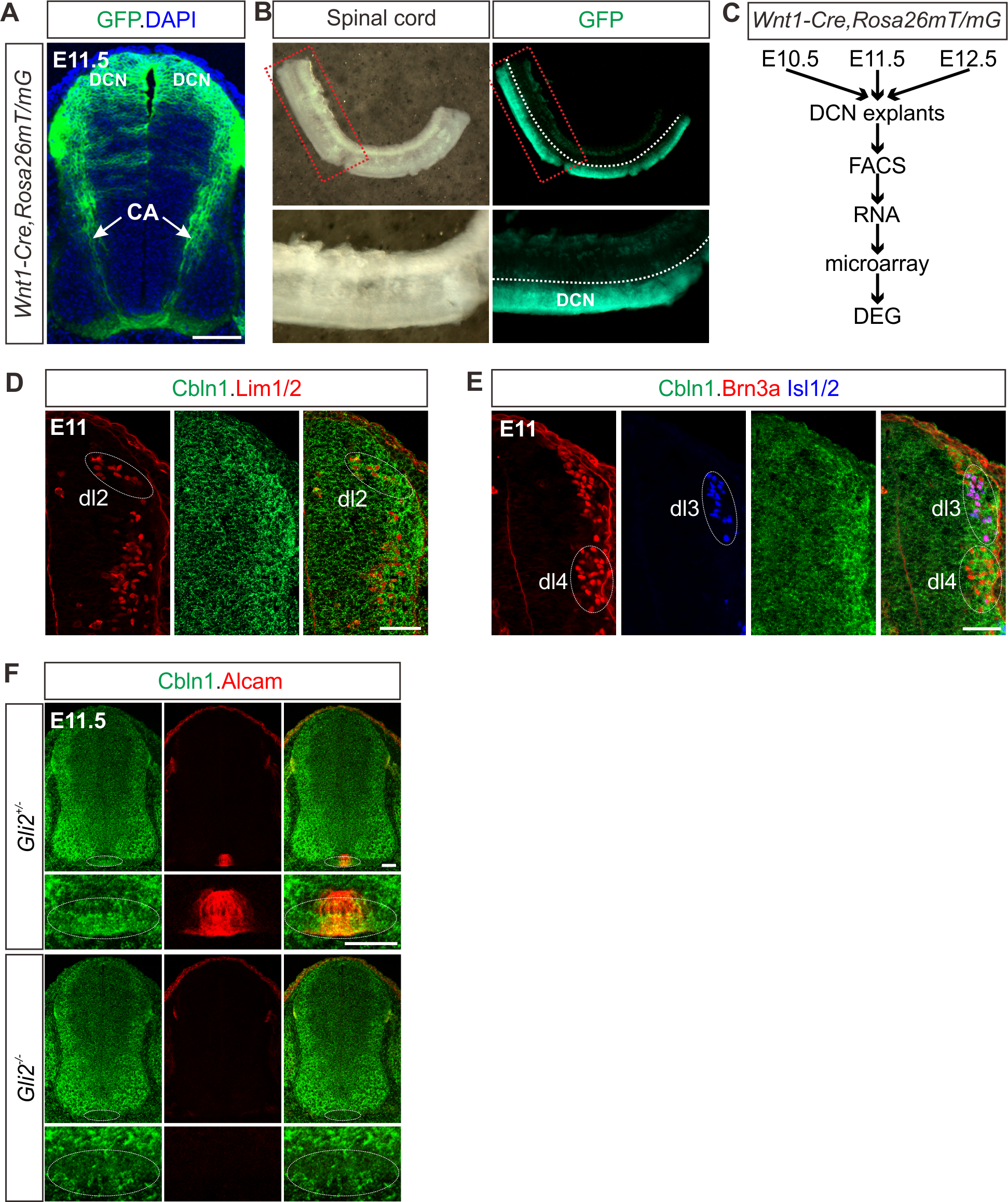

Supplement: S1 Fig — (A) The embryonic dorsal spinal neurons were genetically labeled with eGFP by crossing Wnt1-cre with Rosa26mTmG mice. Immunofluorescence of cross-sections of E11.5 spinal cord was shown. DCN, dorsal commissural neurons. CA, commissural axons. Scale bar, 100 μm. (B) The dissected E11.5 spinal cords were shown in both bright-field and fluorescent images. The regions in the red dotted boxes were shown with higher magnification in the lower images. The dotted white line indicates where to cut and separate dorsal and ventral spinal cord. (C) The scheme showing the procedures for identifying the differentially expressed genes in the mouse embryonic dorsal spinal cord. (D and E) Co-immunostaining of Cbln1 with Lim1/2 (D) or Brn3a and Isl1/2 (E) in spinal cord cross-sections at E11 showed expression of Cbln1 in the DCNs. Circled areas highlight the expression of Cbln1 in the dI2 neurons marked by Lim1/2, the dI3 neurons co-labeled by Brn3a and Isl1/2, and the Brn3a+ dI4 neurons just below dI3. Scale bars, 50 μm. (F) Co-immunostaining of Cbln1 with Alcam in spinal cord cross-sections of Gli2 KO and its littermate control embryos at E11.5. The circled areas indicate the expression and loss of Cbln1 in control and Gli2 KO embryos, respectively. Scale bar, 50 μm. DCN, dorsal commissural neurons; CA, commissural axon; FACS, Fluorescence-activated cell sorting; DEG, differentially expressed genes; dI2, dI3, dI4, dorsal interneuron 2, 3, 4. (TIF) [file pbio.3001853.s001.tif]

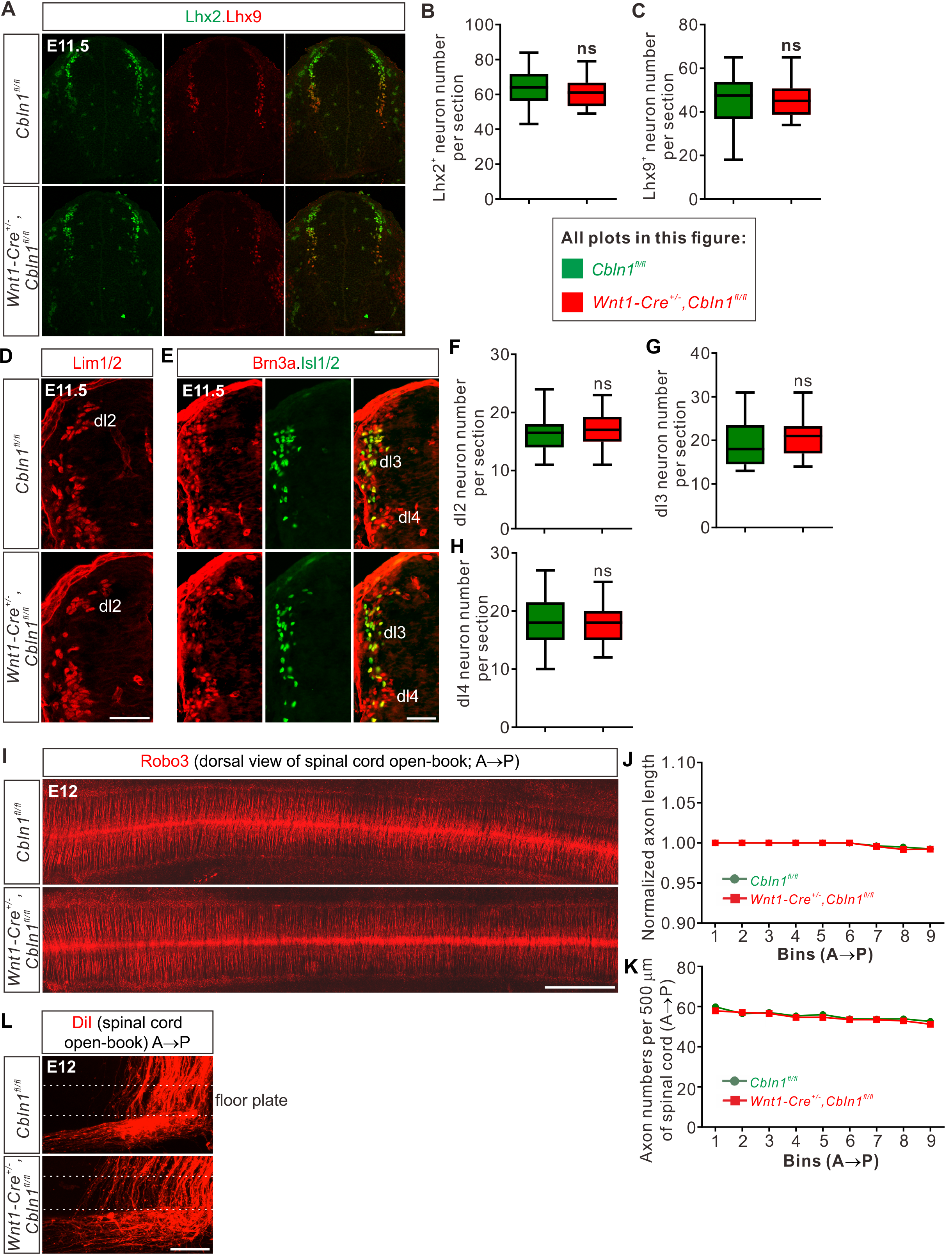

Supplement: S2 Fig — (A) Lhx2 and Lhx9 immunostaining in E11.5 spinal cord indicated that Cbln1 cKO in DCNs does not disturb neurogenesis or patterning of the dI1 neurons. Scale bar, 100 μm. (B and C) Quantification of Lhx2+ and Lhx9+ neurons in (A) showed that the dI1 neurogenesis is not affected in Cbln1 cKO. All data are represented as box and whisker plots: Cbln1fl/fl (n = 20 sections) vs. Wnt1-Cre+/-,Cbln1fl/fl (n = 16 sections); ns, not significant (p = 0.46 for Lhx2+ neurons in B, p = 0.99 for Lhx9+ neurons in C); by unpaired Student t test. (D and E) Lim1/2 and Brn3a/Isl1/2 immunostaining in E11.5 spinal cord indicated that Cbln1 cKO in DCNs does not affect neurogenesis or patterning of the dI2, dI3, or dI4 neurons. Scale bars, 50 μm. (F–H) Quantification of dl2, dl3, and dl4 neurons in (D and E) showed that their neurogenesis is not affected. All data are represented as box and whisker plots: Cbln1fl/fl (n = 36 sections for F, n = 33 sections for G and H) vs. Wnt1-Cre+/-,Cbln1fl/fl (n = 38 sections for F–H); ns, not significant (p = 0.37 for dl2 neurons in F, p = 0.31 for dl3 neurons in G, p = 0.79 for dl4 neurons in H); by unpaired Student t test. (I) Commissural axon growth could catch up at later stages in DCN-specific Cbln1 cKO in vivo. Commissural axons were marked by Robo3 immunostaining in spinal cord open-books at E12, showing that almost all Robo3-labeled commissural axons in Cbln1 cKO reached the floor plate. Scale bar, 500 μm. (J and K) Quantification of commissural axon lengths and numbers in (I). The spinal cords were divided to bins (500 μm) along the anterior-posterior (A➔P) direction. All data are mean ± SEM: Cbln1fl/fl (n = 8 embryos) vs. Wnt1-Cre+/-,Cbln1fl/fl (n = 10 embryos). All bins show no difference in axon length (J) or number (K) by multiple t tests. (L) DiI labeling of spinal cord open-books at E12 showed that crossing or post-crossing commissural axons are behaving normally in DCN-specific Cbln1 cKO embryos compared with controls. Scale bar, 50 μm. T [file pbio.3001853.s002.tif]

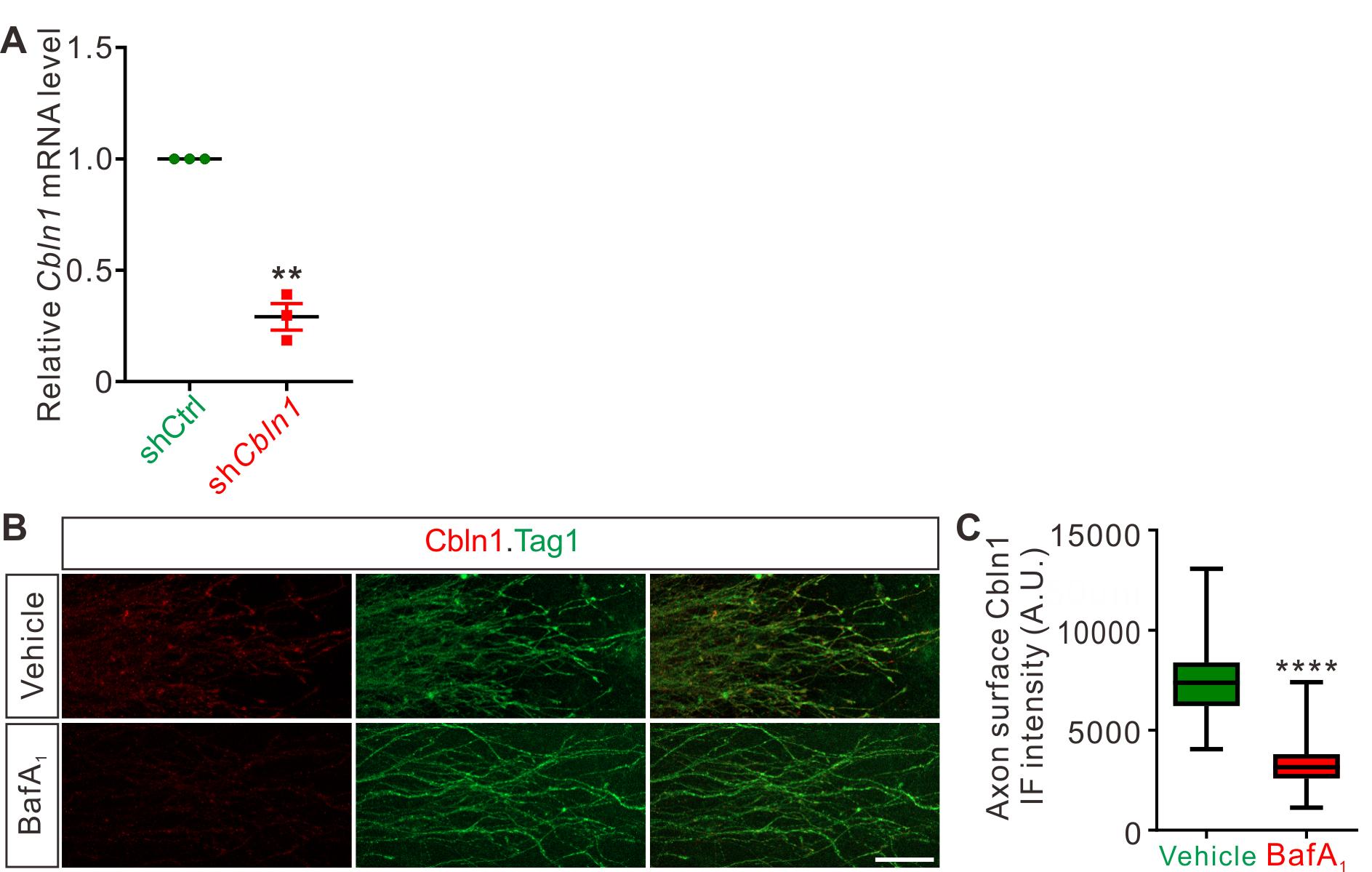

Supplement: S3 Fig — (A) Validation of knockdown of Cbln1 using lentiviral shCbln1. Since Cbln1 is abundant in cerebellar granule cells, we prepared dissociated cerebellar granule cells from P8 mouse pups and cultured in vitro to test the knockdown efficiency of shCbln1. Cbln1 mRNA levels were measured by RT-qPCR after lentiviral shRNA infection. Data are mean ± SEM and represented as dot plots: **p = 0.0070; by unpaired Student t test. (B) Bafilomycin A1 (BafA1) blocked lysosomal exocytosis of Cbln1 from commissural axons. Cbln1 IF signals were reduced on the commissural axon surface of cultured DCN explants after 200 nM BafA1 treatment for 4 h. Scale bar, 50 μm. (C) Quantification of axon surface Cbln1 IF signals in (B). Data are represented as box and whisker plots: Vehicle (n = 182 axons) vs. BafA1 (n = 220 axons), ****p = 5.8 × 10−106, by unpaired Student t test. The data underlying all the graphs shown in the figure are included in S1 Data. Cbln1, cerebellin 1; BafA1, Bafilomycin A1; shCtrl, control shRNA; shCbln1, shRNA against Cbln1; SEM, standard error of the mean; DCNs, dorsal commissural neurons; IF, immunofluorescence; A.U., arbitrary unit. (TIF) [file pbio.3001853.s003.tif]

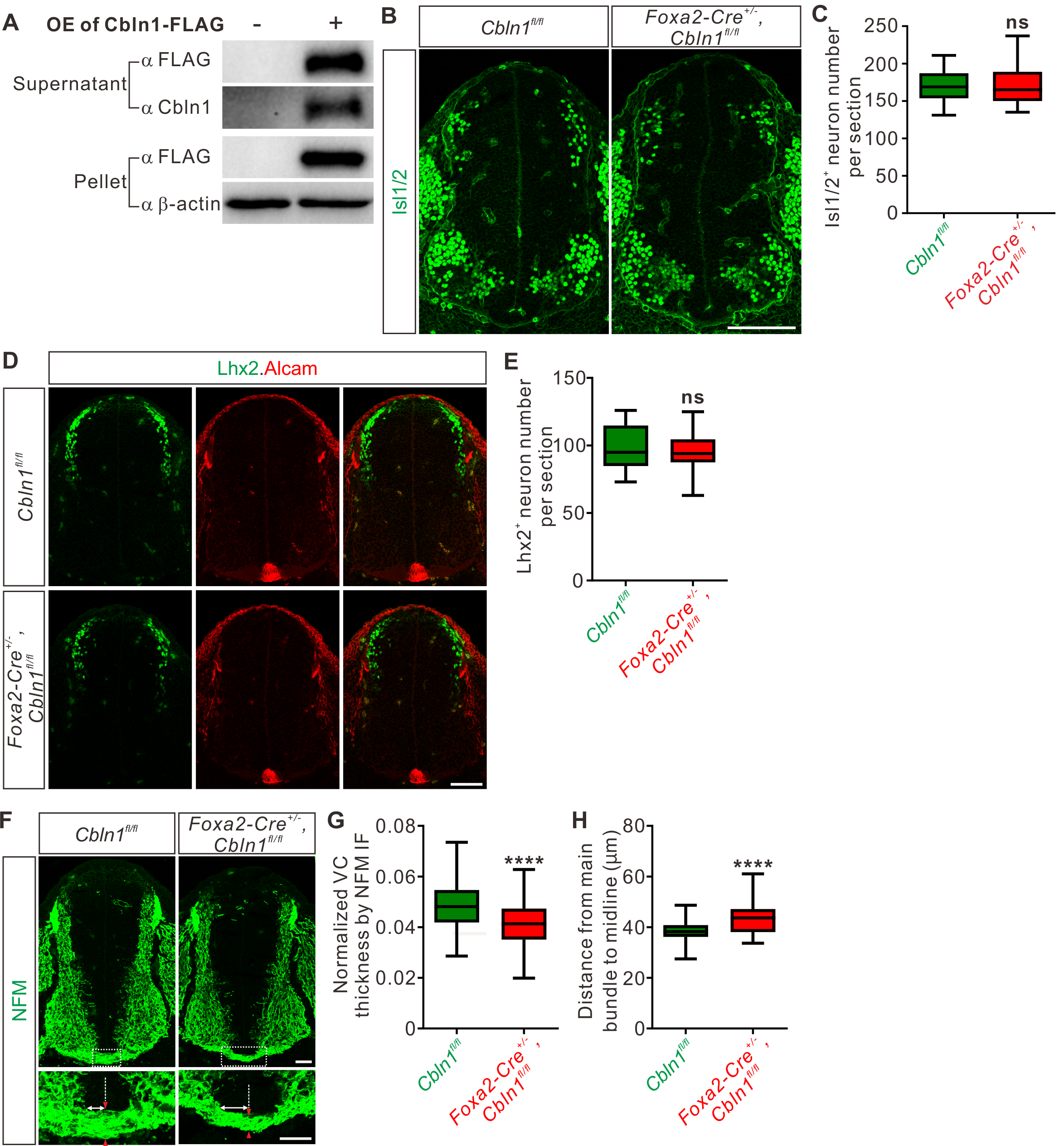

Supplement: S4 Fig — (A) Overexpression and secretion of Cbln1 tagged by FLAG in COS7 cells were validated by WB. (B and C) Isl1/2 immunostaining showed normal patterning of spinal cord in the floor plate-specific Cbln1 cKO embryos (B). Isl1/2 marks different interneurons and motor neurons in spinal cord. The data for quantification of Isl1/2+ neuron numbers are represented as box and whisker plots (C): Cbln1fl/fl (n = 42 sections) vs. Foxa2-Cre+/-,Cbln1fl/fl (n = 49 sections); ns, not significant (p = 0.93); by unpaired Student t test. Scale bar, 100 μm (B). (D and E) The floor plate-specific cKO of Cbln1 does not disturb DCN neurogenesis, spinal cord patterning, or floor plate development. Lhx2 and Alcam immunostainings of E11.5 spinal cord were used to mark dI1 commissural neurons and floor plate, respectively (D). The data for quantification of Lhx2+ neuron numbers are represented as box and whisker plots (E): Cbln1fl/fl (n = 16 sections) vs. Foxa2-Cre+/-,Cbln1fl/fl (n = 35 sections); ns, not significant (p = 0.59); by unpaired Student t test. Scale bar, 100 μm (D). (F) The axon guidance defects of pre-crossing commissural axons were observed by NFM immunostaining in floor plate-specific Cbln1 cKO and control embryos at E11.5. Higher magnification views of the FP region in the white dotted boxes are also shown (bottom). The pair of red arrowheads denotes the thickness of the VC. The double-arrowed line measures the distance between the point of intersection (of the main pre-crossing commissural axon bundle with the ventral edge of spinal cord) and the midline (indicated by the dotted line). Scale bars, 50 μm. (G and H) Quantification of the VC thickness and the distance from the main bundle intersection point to the midline. All data are represented as box and whisker plots: Cbln1fl/fl (n = 49 sections) vs. Foxa2-Cre+/-,Cbln1fl/fl (n = 74 sections), ****p = 3.86 × 10^−5^ for G, ****p = 1.35 × 10^−7^ for H, by unpaired Student t test. The data underlying all the graphs shown in the figur [file pbio.3001853.s004.tif]

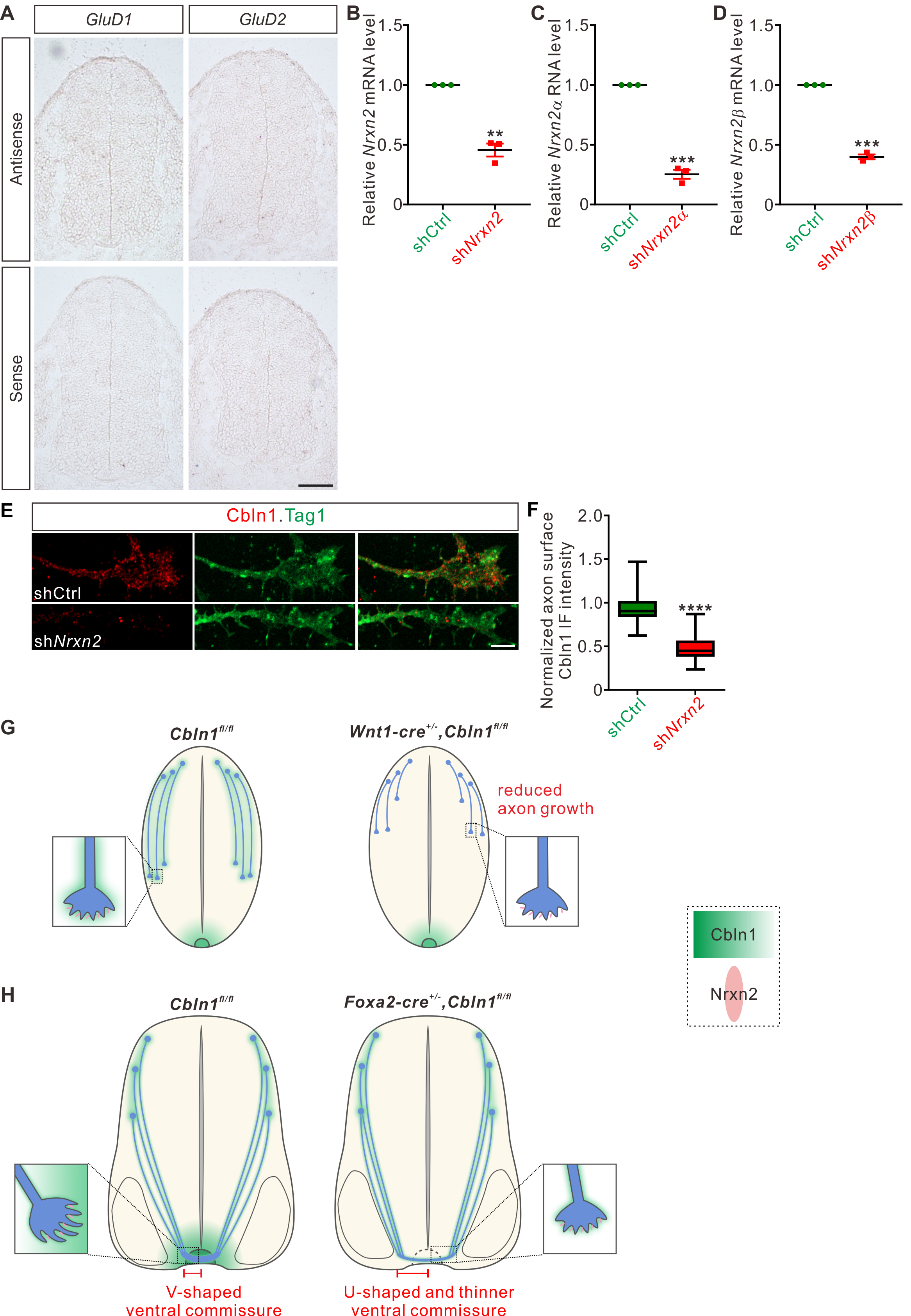

Supplement: S5 Fig — (A) GluD1 or GluD2 mRNA was not detected in E11.5 spinal cord cross-sections by in situ hybridization. Scale bar, 100 μm. (B–D) Validation of knockdown by shRNAs. Dissociated cerebellar granule cells from P8 mouse pups were cultured and lentiviral shRNAs were infected. Significant knockdown was achieved by shRNAs against Nrxn2, Nrxn2α, and Nrxn2β, respectively. RT-qPCR data are mean ± SEM and represented as dot plots: **p = 0.0084 for B; ***p = 0.00010 for C; ***p = 0.00020 for D; by unpaired Student t test. (E) Surface Cbln1 IF signals were reduced in the commissural axons and growth cones after lentiviral shNrxn2 infection. Dissociated DCN neurons from E11 mouse embryos were cultured and infected by lentiviral shNrxn2. Surface Cbln1 IF signals were imaged. Scale bar, 5μm. (F) Quantification of axon surface Cbln1 IF signals in (E). Data are represented as box and whisker plots: shCtrl (n = 27 axons) vs. shNrxn2 (n = 26 axons), ****p = 1.13 × 10^−12^, by unpaired Student t test. (G) Working model for the stimulation of commissural axon growth by the cell-autonomous Cbln1. In the pre-crossing commissural axons, Cbln1 is expressed cell-autonomously by the dorsal commissural neurons and axons. Commissural axon growth cone-secreted Cbln1 works back to itself in an autocrine manner and binds to Nrxn2 receptors to stimulate commissural axon growth. In the DCN-specific Cbln1 cKO embryos, commissural axon growth is reduced compared with their littermate controls. (H) Working model for the attraction of commissural axon growth toward midline by the non-cell-autonomous, floor plate-derived Cbln1. When commissural axons approach the midline, the floor plate-derived Cbln1 attracts commissural axons to the midline that is also mediate by Nrxn2 receptors. In the floor plate-specific Cbln1 cKO embryos, commissural axon guidance in the midline crossing is impaired, resulting in a U-shaped and thinner ventral commissure compared with the V-shaped and thick ventral commissures in the l [file pbio.3001853.s005.tif]

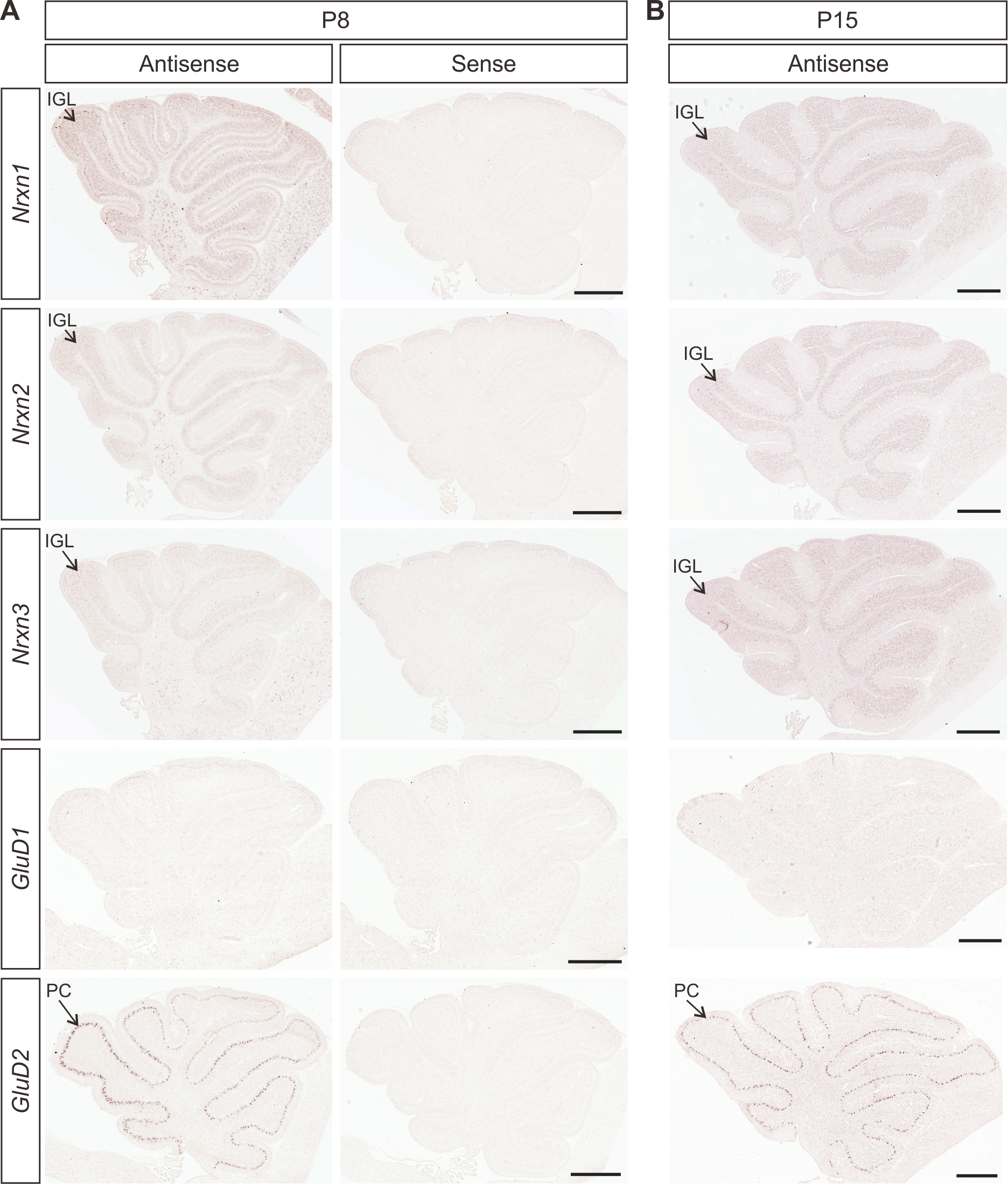

Supplement: S6 Fig — (A and B) In situ hybridization of Nrxn1, Nrxn2, Nrxn3, GluD1, and GluD2 in cerebella at P8 (A) and P15 (B). Nrxn1, Nrxn2, and Nrxn3 mRNAs were detected in the IGL. GluD2 mRNA was highly and specifically expressed in the PCs while GluD1 mRNA was not detected in the cerebellum at these stages. Scale bars, 500 μm. Cbln1, cerebellin 1; Nrxn, neurexin; GluD1, GluD2, glutamate receptor delta 1, 2; IGL, inner granule layer; PC, Purkinje cells. (TIF) [file pbio.3001853.s006.tif]

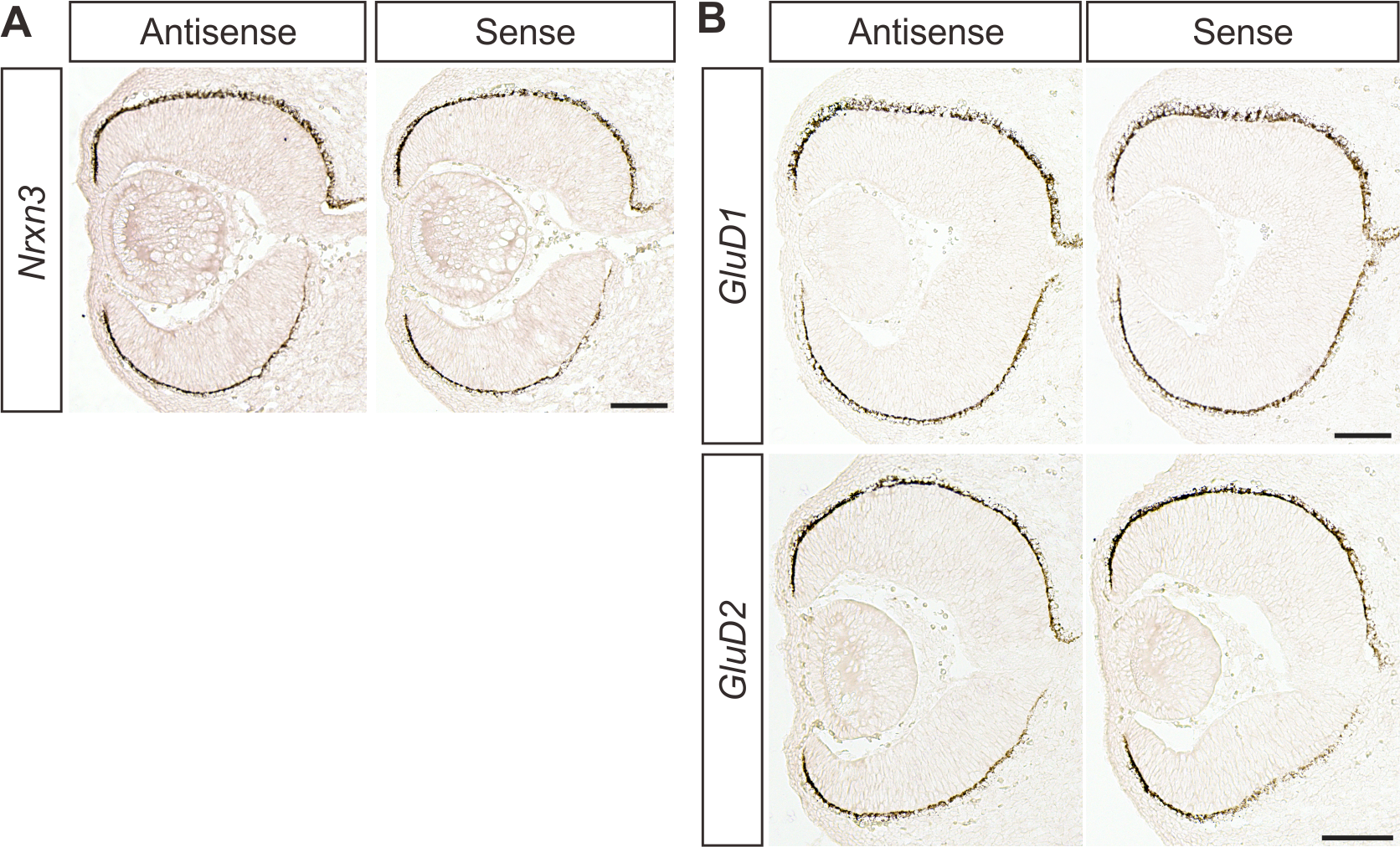

Supplement: S7 Fig — (A and B) In situ hybridization of Nrxn3 (A), and GluD1 and GluD2 (B) in E13 retina. Nrxn3, GluD1, or GluD2 mRNA was not detected in the developing retina. Scale bars, 100 μm. Cbln1, cerebellin 1; Nrxn3, neurexin 3; GluD1, GluD2, glutamate receptor delta 1, 2. (TIF) [file pbio.3001853.s007.tif]

Raw Western blot images for S4A Fig

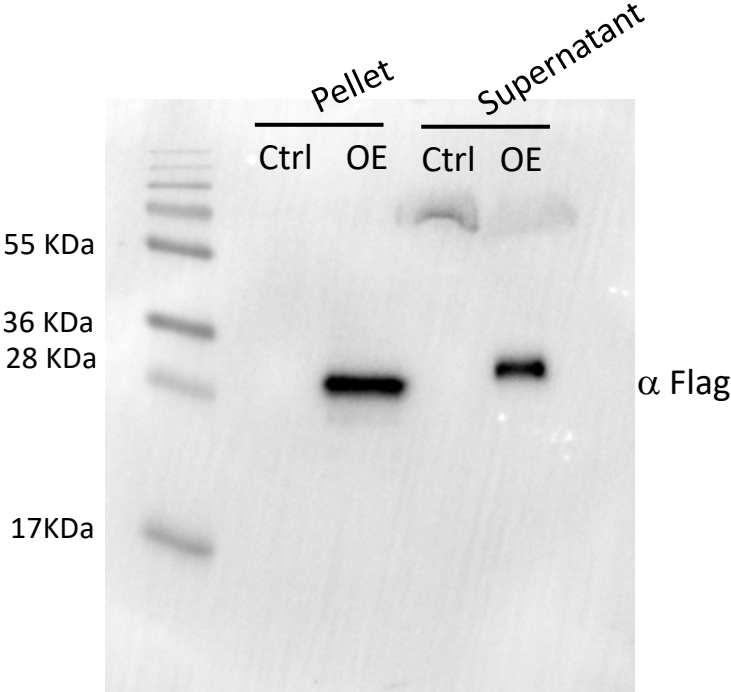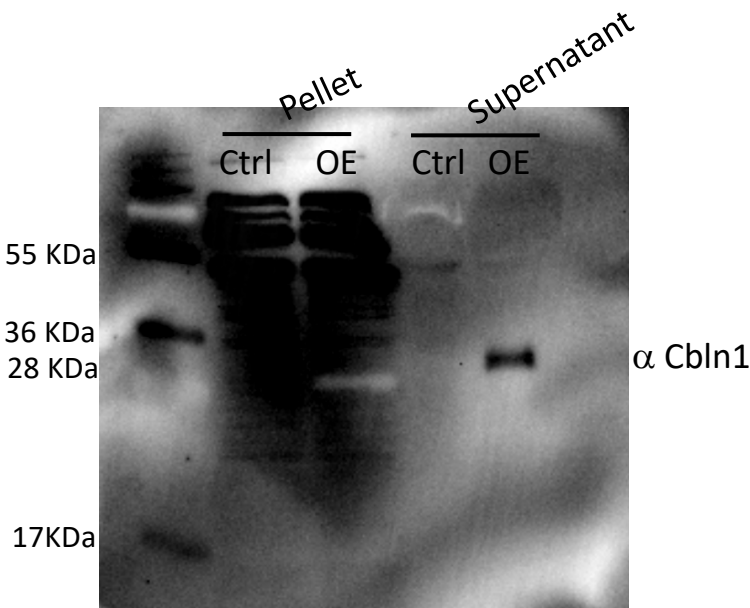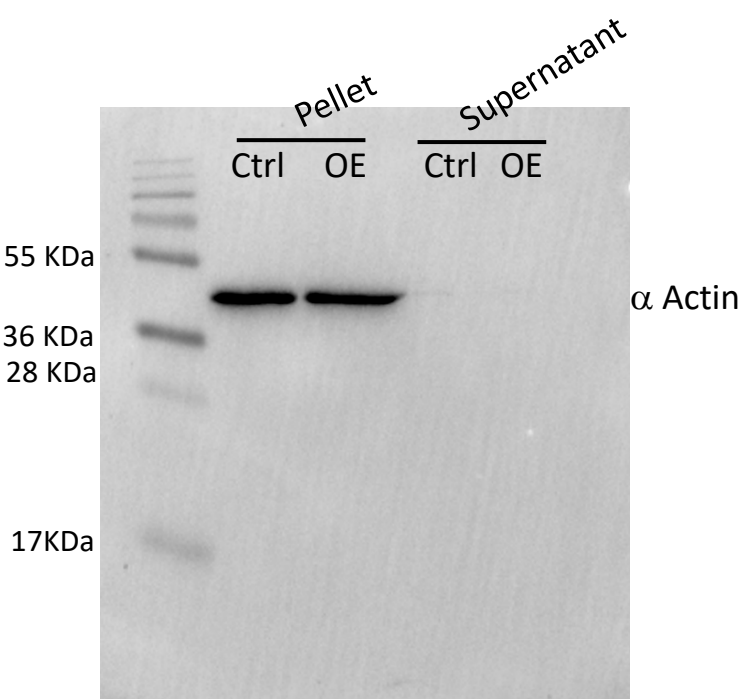

Supplement: S1 Raw Images — (PDF) [file pbio.3001853.s009.pdf]
