# Supplementary material for: Cbln1 regulates axon growth and guidance in multiple neural regions
Source: PLoS Biol. 2022 Nov 17;20(11):e3001853. doi: 10.1371/journal.pbio.3001853 (PMC9671368; doi:10.1371/journal.pbio.3001853)
Supplement: Attachment — Submitted filename: Responses to the reviewers comments_20220807.pdf [file pbio.3001853.s011.pdf]

Responses to the reviewers' comments

Re: PBIOLOGY-D-22-00788R1

Rev. 1:

The authors of this study investigated a novel role for the protein Cerebellin-1 (Cbln1) in axon guidance during development. Using microarray analysis of dorsal spinal cord neurons, Cbln1 was identified as one of the differentially expressed gene candidates and verified by in situ hybridization that its mRNA is expressed both in dorsal spinal commissural neurons and the ventral floor plate. In order to study the cell autonomous (dorsal commissural neurons) and non-cell autonomous (floor plate) roles of Cbln1, a conditional flox Cbln1 mouse was generated and crossed with tissue-specific Cre lines. Using in vivo mouse genetics for loss-of-function, in utero electroporation in the chick for gain-of-function and in vitro tissue culture assays, it was shown that Cbln1 promoted commissural axon growth cell autonomously in commissural neurons and guidance by the floor plate in a non-cell autonomous manner. The investigators go on to show that Cbln1 most likely mediates these axon guidance events through its receptor Neurexin-2. It is also interesting that Cbln1 signaling mediates axonal growth and guidance in other neuronal populations including cerebellar granule cells and retinal ganglionic cells, of which these results suggest that Cbln1 is used widely by the nervous system for axon growth and guidance besides its previously known role in synaptogenesis. This study adds new knowledge to the complex and diverse molecular mechanisms involved in axon guidance during early nervous system development. However, this study falls short of demonstrating the downstream signaling mechanism employed by Cbln1 in axon guidance. It is also not known whether the same or different signaling mechanisms are used by Cbln1 in synaptogenesis versus axon guidance, which would add substantial significance to this work.

Here are a few issues that the authors should address:

1. Higher magnifications images should also be presented of the open-book preparation for the WT and conditional mutant in Fig. 2B (Fig. 2C in the revised version) to better illustrate what the authors claimed that there are fewer number of commissural axons and shorter axons, especially when it is not clear in the quantification shown in Fig. 2D (Fig. 2E in the revised version) whether individual axons or bundled of axons are actually counted.

We thank the reviewer for the suggestion, and we now have shown higher magnification images to better illustrate there are fewer numbers of and shorter commissural axons in the DCN-specific *Cbln1*

cKO spinal cords (revised Fig. 2C' and 2C''). For the quantification shown in the revised Fig. 2E, the individual axons were counted.

2. Fig. 3F and 3H show the in vitro commissural axon cultures treated with GPN to block Cbln1 exocytosis resulting in slower axon growth rate. First, the image panels in 3F does not appear to be different in terms of illustrating axon growth rate. Second, did the investigators look at whether the lack of Cbln1 signaling in the axons, especially in the in vivo conditional knockout mutants, only slow down the growth rate and that the mutant axons will eventually catch-up. This would make sense as there are other attractant and growth promoting molecules used by the dorsal spinal commissural neurons, like Netrin-DCC.

For the first point, we are sorry for the confusion for the reviewer. Fig. 3F (IF with fixed axons) shows that GPN can block Cbln1 exocytosis in axons, which is demonstrated by decreased axon surface Cbln1 IF signals (Fig. 3F and 3G). We did not use Fig. 3F to show slower axon growth rate since these axons are fixed. For measuring axon growth rate, we imaged the live axons at two time points (0 and 7 h) in live explant cultures with vehicle or GPN treatment, and then calculated axon growth rates (Fig. 3H).

For the second point, we agree that other cell-autonomous growth promoting molecules or non-cell-autonomous guidance cues (including Cbln1 from floor plate) are not affected in the DCN-specific *Cbln1* cKO embryonic spinal cord. As suggested, we continued to check whether the commissural axon growth in the DCN-specific *Cbln1* cKO could eventually reach the floor plate. As shown in S2I Fig, almost all axons reached the floor plate at E12, and there was no difference between DCN-specific *Cbln1* cKO and control for commissural axon length or number at this stage (S2J and S2K Fig). These data suggest that the DCN-specific *Cbln1* cKO axons will eventually catch up.

3. While this reviewer appreciates the brevity of the Discussion, in this case it might be a bit lacking. There is no discussion or speculation as to why Cbln1-Neurexin 2 is required by the dorsal commissural axons or other neurons and their axons for promoting growth and guidance when there are already known attractants and growth factors doing the same job. Is Cbln1-Neurexin 2 required for a specific subset of commissural axons as there are at least 4 different types, dI1-dI4, in the dorsal rodent spinal cord during development.

We agree with the reviewer that it is surprising that Cbln1-Neurexin 2 which has been shown to work as synaptic organizers is also required by the dorsal commissural neurons and other neurons for promoting growth and guidance when there are already known attractants and growth factors doing the same job. However, quite a few studies have shown that these synaptic organizers are also involved in earlier neural development including axon development. In *C. elegans*, Nrxn promotes neurite outgrowth of DVB neurons [1], and a neurexin-related protein, BAM-2, regulates axonal branches [2]. In *Drosophila*, homolog of α-neurexin (DNrx) restricts axonal branching [3], and neurexin and neuroligin-based adhesion molecules regulate axonal arborization growth independent of synaptic activity [4]. These studies suggest that these synapse organizers are also required in early neural development to regulate axon development, working together with those already known attractants and growth factors.

And vice versa, signaling pathways mediated by the known axon guidance cues and growth factors have also been shown to regulate synaptogenesis and synaptic plasticity, such as Netrin-DCC [5-8], Slit-Robo [9-12], Semaphorin-neuropilin-plexin [13-26], Wnt-Frizzled [27-39], FGF-FGFR [39-43], BMP/TGFβ signaling [37-39, 44-65], neurotrophin-Trk [66-75], and GDNF-GFR [76-79].

Together, these findings suggest that both axon guidance cues & growth factors and synaptic organizers have dual roles in both earlier axon development and later synapse organization.

We thank the reviewer for asking if Cbln1 is only required for a specific subset of commissural axons as there are at least 4 different types, dI1-dI4, in the dorsal rodent spinal cord during development. Co-immunostaining of Cbln1 with Lhx2, a dI1 DCN marker [80], confirmed the expression of Cbln1 in the dI1 DCNs of developing spinal cords from E10.5 to E12.5 (Fig 1B-1D). We continued to find that expression of Cbln1 was also detected in dI2-4 DCN subpopulations (revised S1D and S1E Fig). These data suggest that Cbln1 might regulate commissural axon development in all dI1-dI4 DCN neurons.

4. Finally, a minor correction is needed on Page 13 in the subsection "Cbln1-Nrxn signaling in axon development", first sentence: "esp." needs to be spelled out.

We thank the reviewer for pointing out this and have corrected it now.

Rev. 2:

This manuscript by Han et al., identifies Cerebellin-1 as an attractive guidance cue for axons from several populations of neurons, including dorsal commissural neurons, retinal ganglion cells, and cerebellar granule neurons. Identification of a novel guidance cue is significant to the field. Previously, Cerebellin-1 had only been examined in synapse formation. Here, the authors use both mouse and chick models to demonstrate a role for Cerebellin-1 in axon guidance. They find evidence for both cell autonomous functions of the protein (promoting outgrowth in dorsal commissural neurons), as well as non-cell autonomous roles of Cerebellin-1 secretion from the floor plate and the ventral diencephalon to guide axons. Overall, the manuscript convincingly defines such a broad and conserved role. I have one suggested experiment, and several points for clarification.

1. My single experimental suggestion would be to more fully parse the cell autonomous and non-autonomous rolls of cerebellin-1 for DCNs. When Cerebellin-1 is specifically knocked out of DCNs, do they ever reach the floor plate, or do they not grow long enough to get there? Or what happens in an open book DiI assay?

We thank the reviewer for the suggestion. As suggested, we continued to check whether the commissural axon growth in the DCN-specific *Cbln1* cKO embryos could eventually reach the floor plate. As shown in S2I Fig, almost all axons reached the floor plate at E12, and there was no difference between DCN-specific *Cbln1* cKO and control for commissural axon length or number at this stage (S2J and S2K Fig). These data suggest that the DCN-specific *Cbln1* cKO axons will eventually catch up.

We also performed the open book DiI assay at E12, and the crossing or post-crossing behaviors of commissural axons in the DCN-specific *Cbln1* cKO embryos were also similar to those of controls (S2L Fig).

These results are not surprising since other cell-autonomous growth promoting molecules or non-cell-autonomous guidance cues (including Cbln1 from floor plate) are not affected in the DCN-specific *Cbln1* cKO embryonic spinal cord.

Clarification Points:

1. At the beginning of the results, "differentially expressed genes" needs to be clarified by adding "over developmental time." As written now, it is quite vague.

We thank the reviewer for the suggestion, and we now have revised this in the manuscript.

2. Figure 1A: Motor neurons should also be highlighted in E12.5.

For Fig. 1A, as suggested, we now have highlighted Cbln1 expression in motor neurons at E11.5 and E12.5.

3. Figure 1B-D: Not really convinced by the LHx2 staining that Cbln1 is expressed in DCN (Figure 2A (Fig. 2B in the revised version) is more convincing). Adding zoomed insets may help?

We thank the reviewer for the suggestion, and we now have shown the higher magnification insets to highlight the expression of Cbln1 in $Lhx2^+$ dI1 DCN neurons (Fig. 1B'-1D').

4. Figure 3A-C: Old work from Tessier-Lavigne group showed no outgrowth of spinal explants, unless netrin was present. Was a modification to your system made? Commenting on this would be helpful.

Both the purpose and the design of our DCN explant culture experiments are different from those of Tessier-Lavigne group. We wanted to test if Cbln1 could regulate commissural axon growth. However, Netrin1 could promote axon growth, which may cover the effect of Cbln1. So we could not add Netrin1 in our study. Tessier-Lavigne group used Netrin1 to coat plate and did the culture for 16 h [81]. We use collagen gel and culture the explants for 2 days: there are indeed few axons growing in 24 h; however, after 40 h, explants have lots of axons growing out.

3F-H: worth using an additional inhibitor to nail this down a role for the lysosome or otherwise more clearly citing role for lysosome exocytosis in cerebellin-1 secretion from other papers for those not seeped in the field.?

We thank the reviewer for the suggestion. It has been reported that Cbln1 co-localizes with the lysosomal enzymes cathepsin B and D in the adult mouse brain [82, 83], indicating the lysosome may regulate Cbln1 secretion in commissural axons. To test this, we applied different lysosome inhibitors to the DCN cultures and checked their effects on Cbln1 secretion from commissural axons. In addition to GPN, we also used Bafilomycin A1 ($BafA_1$), which blocks lysosomal functions through working as a specific inhibitor of vacuolar H^+-ATPase [84]. Treatment of DCN cultures with GPN or $BafA_1$, followed

by an IF protocol to detect surface Cbln1 by leaving out the permeabilization steps, showed loss of Cbln1 IF signals on the commissural axon surface (Fig 3F and 3G; revised S3B and S3C Fig), suggesting that Cbln1 is released from lysosomes in commissural axons and growth cones.

5. Figure 6F (now removed in the revised version): clarify in the text that this addition to wildtype, although would be interesting if rescued in knockout?

We thank the reviewer for the suggestion, and we now have performed the rescue experiment to replace the old data (original Fig. 6F is now removed in the revised version). Axon growth rates of Cbln1-deficient GCs *in vitro* were significantly decreased compared with control neurons (revised Fig 6D and 6E), suggesting that the cell-autonomous Cbln1 is required for GC axon growth. Similar to Cbln1 on commissural axons, extrinsic application of the recombinant hCbln1 (rhCbln1) protein to the GC axons could efficiently rescue this axon growth defect (revised Fig 6D and 6E).

6. Figure 7C: Tag1 is marking axons of RGC---how were the regions defined, as it is unclear.

Optic chiasm forms a specific shape [85, 86], where Tag1^{+} RGC axons traverse the chiasm region and enter the optic tracts in the lateral wall of the diencephalon [87]. We used Tag1 to mark the RGC axons navigating through this part. We now have removed the lines highlighting Tag1 IF signals.

7. Figure 7D: Please include representative images.

We now have included the representative images for the axon guidance assay using retinal explants (revised Fig. 7D), and the quantification data were also updated (revised Fig. 7E).

8. Figure 7H (Fig. 7I in the revised version): Why don't we see ipsilateral projection here? My understanding is that DiI labeling would also label the ipsilateral axons.

This DiI tracing experiment was performed at E13 (revised Fig. 7I). Ipsilateral RGC neurogenesis in retina starts to increase at E13. It is hard to observe the ipsilateral axons at E13 since there are only a few axons which just reach the optic chiasm and start to turn to the ipsilateral side [88]. Our purpose is to check the guidance of contralateral RGCs (Brn3a^{+}Nrxn2^{+}), so we checked the optic chiasm at the beginning timepoint (E13), and continued to check the terminal projection in LGN at P4 (revised Fig. 7K and 7L).

Other suggestions:

* Ibata et al shows that secreted Cbln1 binds Neurexin and diffuses along the axon. The authors show IF images of Cbln1 (Fig 3) and Neurexin (Fig 5) each localizing to commissural axons and the growth cone in cultured DCN neurons. May be useful to show if these proteins co-localize at the axon/growth cone in cultured neurons? Furthermore, does loss of Neurexin in cultured neurons decrease Cbln1 staining at the axon/growth cone?

We thank the reviewer for suggesting these interesting experiments. We could not show the co-localization of Cbln1 and Nrxn2 at the axon/growth cone in the cultured neurons because both Cbln1 and Nrxn2 antibodies that we tried to get working in the lab are from rabbit. As suggested, we checked surface Cbln1 distribution after Nrxn2 knockdown in culture neurons. We found that axonal surface Cbln1 signals were decreased after Nrxn2 knockdown in culture DCN neurons (revised S5E and S5F Fig).

* In the discussion, I would be interested in hearing the authors' thoughts on the other Cerebellin isoforms/any possible redundancy (or lack thereof) in axon guidance.

Cbln1 and Cbln2 bind to GluRδ2 and Nrxn1-3, while Cbln4 is a highly specific ligand for DCC [89]. In addition, *Cbln4*-null mice did not show any defect in commissural axon guidance in the developing spinal cord [89].

Cbln3 has only been studied in synapse structural and functional regulation. Cbln3 is secreted only when it is bound to Cbln1 [90]. Cbln1 and Cbln3 have been shown to reciprocally regulate each other's degradation and secretion, so that Cbln1 and Cbln3 proteins are both lost in *Cbln1*-null mice, while *Cbln3*-null mice lack Cbln3 but have a robust increase in Cbln1 protein level [90]. Consequently, *Cbln1* and *Cbln3* single knockout mice have different phenotypes while *Cbln1*-*Cbln3* double mutant mice have deficits identical to those of *Cbln1*-null mice [90]. It would be interesting to check if Cbln3 is expressed in the developing dorsal spinal cord and brain, and if there is a similar Cbln1-Cbln3 interaction mechanism in the earlier embryonic stages.

It has been reported that Cbln1 and Cbln2 have redundancy in cerebellum but not in thalamic neurons in the adult mice [91], supporting a tissue-specific redundancy in adulthood. Cbln2 is expressed in the developing chick dorsal spinal cord [92], and our own *in situ* hybridization result also

shows Cbln2 expression in the developing mouse dorsal spinal cord (unpublished data). *Cbln1-Cbln2* double cKO may be useful to check their redundancy in the developing brain regions and axon developmental processes described in our current study.

References

1. Hart MP, Hobert O. Neurexin controls plasticity of a mature, sexually dimorphic neuron. Nature. 2018;553(7687):165-70. Epub 2018/01/13. doi: 10.1038/nature25192. PubMed PMID: 29323291; PubMed Central PMCID: PMCPMC5968453.
2. Colavita A, Tessier-Lavigne M. A Neurexin-related protein, BAM-2, terminates axonal branches in C. elegans. Science. 2003;302(5643):293-6. Epub 2003/10/11. doi: 10.1126/science.1089163. PubMed PMID: 14551437.
3. Liu L, Tian Y, Zhang XY, Zhang X, Li T, Xie W, et al. Neurexin Restricts Axonal Branching in Columns by Promoting Ephrin Clustering. Dev Cell. 2017;41(1):94-106 e4. Epub 2017/04/04. doi: 10.1016/j.devcel.2017.03.004. PubMed PMID: 28366281.
4. Constance WD, Mukherjee A, Fisher YE, Pop S, Blanc E, Toyama Y, et al. Neurexin and Neuroligin-based adhesion complexes drive axonal arborisation growth independent of synaptic activity. Elife. 2018;7. Epub 2018/03/06. doi: 10.7554/eLife.31659. PubMed PMID: 29504935; PubMed Central PMCID: PMCPMC5869020.
5. Glasgow SD, Ruthazer ES, Kennedy TE. Guiding synaptic plasticity: Novel roles for netrin-1 in synaptic plasticity and memory formation in the adult brain. J Physiol. 2021;599(2):493-505. Epub 2020/02/06. doi: 10.1113/jp278704. PubMed PMID: 32017127.
6. Zhou X, Gueydan M, Jospin M, Ji T, Valfort A, Pinan-Lucarré B, et al. The netrin receptor UNC-40/DCC assembles a postsynaptic scaffold and sets the synaptic content of GABA(A) receptors. Nat Commun. 2020;11(1):2674. Epub 2020/05/31. doi: 10.1038/s41467-020-16473-5. PubMed PMID: 32471987; PubMed Central PMCID: PMCPMC7260190.
7. Goldman JS, Ashour MA, Magdesian MH, Tritsch NX, Harris SN, Christofi N, et al. Netrin-1 promotes excitatory synaptogenesis between cortical neurons by initiating synapse assembly. J Neurosci. 2013;33(44):17278-89. Epub 2013/11/01. doi: 10.1523/jneurosci.1085-13.2013. PubMed PMID: 24174661; PubMed Central PMCID: PMCPMC6618363.
8. Glasgow SD, Labrecque S, Beamish IV, Aufmkolk S, Gibon J, Han D, et al. Activity-Dependent Netrin-1 Secretion Drives Synaptic Insertion of GluA1-Containing AMPA Receptors in the Hippocampus. Cell Rep. 2018;25(1):168-82 e6. Epub 2018/10/04. doi: 10.1016/j.celrep.2018.09.028. PubMed PMID: 30282026.
9. Blockus H, Rolotti SV, Szoboszlay M, Peze-Heidsieck E, Ming T, Schroeder A, et al. Synaptogenic activity of the axon guidance molecule Robo2 underlies hippocampal circuit function. Cell Rep. 2021;37(3):109828. Epub 2021/10/24. doi: 10.1016/j.celrep.2021.109828. PubMed PMID: 34686348; PubMed Central PMCID: PMCPMC8605498.
10. Sales EC, Heckman EL, Warren TL, Doe CQ. Regulation of subcellular dendritic synapse specificity by axon guidance cues. Elife. 2019;8. Epub 2019/04/24. doi: 10.7554/eLife.43478. PubMed PMID: 31012844; PubMed Central PMCID: PMCPMC6499537.
11. Campbell DS, Stringham SA, Timm A, Xiao T, Law MY, Baier H, et al. Slit1a inhibits retinal ganglion cell arborization and synaptogenesis via Robo2-dependent and -independent pathways. Neuron.

2007;55(2):231-45. Epub 2007/07/21. doi: 10.1016/j.neuron.2007.06.034. PubMed PMID: 17640525.
12. Ke C, Gao F, Tian X, Li C, Shi D, He W, et al. Slit2/Robo1 Mediation of Synaptic Plasticity Contributes to Bone Cancer Pain. Mol Neurobiol. 2017;54(1):295-307. Epub 2016/01/08. doi: 10.1007/s12035-015-9564-9. PubMed PMID: 26738857.
13. Du Y, Shi Y, Wang X, Song H, Wang X, Hao Y, et al. Hippocampal semaphorin 3B improves depression-like behaviours in mice by upregulating synaptic plasticity and inhibiting neuronal apoptosis. J Neurochem. 2022. Epub 2022/07/28. doi: 10.1111/jnc.15680. PubMed PMID: 35892177.
14. Carulli D, de Winter F, Verhaagen J. Semaphorins in Adult Nervous System Plasticity and Disease. Front Synaptic Neurosci. 2021;13:672891. Epub 2021/05/29. doi: 10.3389/fnsyn.2021.672891. PubMed PMID: 34045951; PubMed Central PMCID: PMCPMC8148045.
15. Jitsuki-Takahashi A, Jitsuki S, Yamashita N, Kawamura M, Abe M, Sakimura K, et al. Activity-induced secretion of semaphorin 3A mediates learning. Eur J Neurosci. 2021;53(10):3279-93. Epub 2021/03/28. doi: 10.1111/ejn.15210. PubMed PMID: 33772906; PubMed Central PMCID: PMCPMC8252788.
16. Frias CP, Liang J, Bresser T, Scheefhals L, van Kesteren M, van Dorland R, et al. Semaphorin4D Induces Inhibitory Synapse Formation by Rapid Stabilization of Presynaptic Boutons via MET Coactivation. J Neurosci. 2019;39(22):4221-37. Epub 2019/03/28. doi: 10.1523/jneurosci.0215-19.2019. PubMed PMID: 30914448; PubMed Central PMCID: PMCPMC6538862.
17. Tan C, Lu NN, Wang CK, Chen DY, Sun NH, Lyu H, et al. Endothelium-Derived Semaphorin 3G Regulates Hippocampal Synaptic Structure and Plasticity via Neuropilin-2/PlexinA4. Neuron. 2019;101(5):920-37 e13. Epub 2019/01/28. doi: 10.1016/j.neuron.2018.12.036. PubMed PMID: 30685224.
18. McDermott JE, Goldblatt D, Paradis S. Class 4 Semaphorins and Plexin-B receptors regulate GABAergic and glutamatergic synapse development in the mammalian hippocampus. Mol Cell Neurosci. 2018;92:50-66. Epub 2018/07/08. doi: 10.1016/j.mcn.2018.06.008. PubMed PMID: 29981480; PubMed Central PMCID: PMCPMC6191356.
19. Acker DWM, Wong I, Kang M, Paradis S. Semaphorin 4D promotes inhibitory synapse formation and suppresses seizures in vivo. Epilepsia. 2018;59(6):1257-68. Epub 2018/05/26. doi: 10.1111/epi.14429. PubMed PMID: 29799628; PubMed Central PMCID: PMCPMC5990477.
20. Wang Q, Chiu SL, Koropouli E, Hong I, Mitchell S, Easwaran TP, et al. Neuropilin-2/PlexinA3 Receptors Associate with GluA1 and Mediate Sema3F-Dependent Homeostatic Scaling in Cortical Neurons. Neuron. 2017;96(5):1084-98 e7. Epub 2017/11/21. doi: 10.1016/j.neuron.2017.10.029. PubMed PMID: 29154130; PubMed Central PMCID: PMCPMC5726806.
21. Uesaka N, Kano M. Presynaptic Mechanisms Mediating Retrograde Semaphorin Signals for Climbing Fiber Synapse Elimination During Postnatal Cerebellar Development. Cerebellum. 2018;17(1):17-22. Epub 2017/10/02. doi: 10.1007/s12311-017-0888-z. PubMed PMID: 28965326.
22. Orr BO, Fetter RD, Davis GW. Retrograde semaphorin-plexin signalling drives homeostatic synaptic plasticity. Nature. 2017;550(7674):109-13. Epub 2017/09/28. doi: 10.1038/nature24017. PubMed PMID: 28953869; PubMed Central PMCID: PMCPMC5907800.
23. Telley L, Cadilhac C, Cioni JM, Saywell V, Jahannault-Talignani C, Huettl RE, et al. Dual Function of NRP1 in Axon Guidance and Subcellular Target Recognition in Cerebellum. Neuron. 2016;91(6):1276-91. Epub 2016/09/13. doi: 10.1016/j.neuron.2016.08.015. PubMed PMID: 27618676.
24. Duan Y, Wang SH, Song J, Mironova Y, Ming GL, Kolodkin AL, et al. Semaphorin 5A inhibits synaptogenesis in early postnatal- and adult-born hippocampal dentate granule cells. Elife. 2014;3.

Epub 2014/10/15. doi: 10.7554/eLife.04390. PubMed PMID: 25313870; PubMed Central PMCID: PMCPMC4236683.
25. Uesaka N, Uchigashima M, Mikuni T, Nakazawa T, Nakao H, Hirai H, et al. Retrograde semaphorin signaling regulates synapse elimination in the developing mouse brain. Science. 2014;344(6187):1020-3. Epub 2014/05/17. doi: 10.1126/science.1252514. PubMed PMID: 24831527.
26. Koropouli E, Kolodkin AL. Semaphorins and the dynamic regulation of synapse assembly, refinement, and function. Curr Opin Neurobiol. 2014;27:1-7. Epub 2014/03/07. doi: 10.1016/j.conb.2014.02.005. PubMed PMID: 24598309; PubMed Central PMCID: PMCPMC4122587.
27. Teo S, Salinas PC. Wnt-Frizzled Signaling Regulates Activity-Mediated Synapse Formation. Front Mol Neurosci. 2021;14:683035. Epub 2021/07/02. doi: 10.3389/fnmol.2021.683035. PubMed PMID: 34194299; PubMed Central PMCID: PMCPMC8236581.
28. Hall AC, Lucas FR, Salinas PC. Axonal remodeling and synaptic differentiation in the cerebellum is regulated by WNT-7a signaling. Cell. 2000;100(5):525-35. Epub 2000/03/18. doi: 10.1016/s0092-8674(00)80689-3. PubMed PMID: 10721990.
29. Cerpa W, Godoy JA, Alfaro I, Farías GG, Metcalfe MJ, Fuentealba R, et al. Wnt-7a modulates the synaptic vesicle cycle and synaptic transmission in hippocampal neurons. J Biol Chem. 2008;283(9):5918-27. Epub 2007/12/22. doi: 10.1074/jbc.M705943200. PubMed PMID: 18096705.
30. Davis EK, Zou Y, Ghosh A. Wnts acting through canonical and noncanonical signaling pathways exert opposite effects on hippocampal synapse formation. Neural Dev. 2008;3:32. Epub 2008/11/07. doi: 10.1186/1749-8104-3-32. PubMed PMID: 18986540; PubMed Central PMCID: PMCPMC2596118.
31. Gogolla N, Galimberti I, Deguchi Y, Caroni P. Wnt signaling mediates experience-related regulation of synapse numbers and mossy fiber connectivities in the adult hippocampus. Neuron. 2009;62(4):510-25. Epub 2009/05/30. doi: 10.1016/j.neuron.2009.04.022. PubMed PMID: 19477153.
32. Sahores M, Gibb A, Salinas PC. Frizzled-5, a receptor for the synaptic organizer Wnt7a, regulates activity-mediated synaptogenesis. Development. 2010;137(13):2215-25. Epub 2010/06/10. doi: 10.1242/dev.046722. PubMed PMID: 20530549; PubMed Central PMCID: PMCPMC2882138.
33. Varela-Nallar L, Alfaro IE, Serrano FG, Parodi J, Inestrosa NC. Wingless-type family member 5A (Wnt-5a) stimulates synaptic differentiation and function of glutamatergic synapses. Proc Natl Acad Sci U S A. 2010;107(49):21164-9. Epub 2010/11/19. doi: 10.1073/pnas.1010011107. PubMed PMID: 21084636; PubMed Central PMCID: PMCPMC3000271.
34. Ciani L, Boyle KA, Dickins E, Sahores M, Anane D, Lopes DM, et al. Wnt7a signaling promotes dendritic spine growth and synaptic strength through Ca^{2+}/Calmodulin-dependent protein kinase II. Proc Natl Acad Sci U S A. 2011;108(26):10732-7. Epub 2011/06/15. doi: 10.1073/pnas.1018132108. PubMed PMID: 21670302; PubMed Central PMCID: PMCPMC3127879.
35. Rosso SB, Inestrosa NC. WNT signaling in neuronal maturation and synaptogenesis. Front Cell Neurosci. 2013;7:103. Epub 2013/07/13. doi: 10.3389/fncel.2013.00103. PubMed PMID: 23847469; PubMed Central PMCID: PMCPMC3701138.
36. Dickins EM, Salinas PC. Wnts in action: from synapse formation to synaptic maintenance. Front Cell Neurosci. 2013;7:162. Epub 2013/11/14. doi: 10.3389/fncel.2013.00162. PubMed PMID: 24223536; PubMed Central PMCID: PMCPMC3819050.
37. Salinas PC. Synaptogenesis: Wnt and TGF-beta take centre stage. Curr Biol. 2003;13(2):R60-2. Epub 2003/01/28. doi: 10.1016/s0960-9822(02)01429-x. PubMed PMID: 12546806.
38. Packard M, Mathew D, Budnik V. Wnts and TGF beta in synaptogenesis: old friends signalling at new places. Nat Rev Neurosci. 2003;4(2):113-20. Epub 2003/02/04. doi: 10.1038/nrn1036. PubMed PMID:

12563282; PubMed Central PMCID: PMCPMC3503525.
39. Salinas PC. Signaling at the vertebrate synapse: new roles for embryonic morphogens? J Neurobiol. 2005;64(4):435-45. Epub 2005/07/26. doi: 10.1002/neu.20159. PubMed PMID: 16041761.
40. Umemori H, Linhoff MW, Ornitz DM, Sanes JR. FGF22 and its close relatives are presynaptic organizing molecules in the mammalian brain. Cell. 2004;118(2):257-70. Epub 2004/07/21. doi: 10.1016/j.cell.2004.06.025. PubMed PMID: 15260994.
41. Dabrowski A, Terauchi A, Strong C, Umemori H. Distinct sets of FGF receptors sculpt excitatory and inhibitory synaptogenesis. Development. 2015;142(10):1818-30. Epub 2015/05/01. doi: 10.1242/dev.115568. PubMed PMID: 25926357; PubMed Central PMCID: PMCPMC4440923.
42. Terauchi A, Johnson-Venkatesh EM, Toth AB, Javed D, Sutton MA, Umemori H. Distinct FGFs promote differentiation of excitatory and inhibitory synapses. Nature. 2010;465(7299):783-7. Epub 2010/05/28. doi: 10.1038/nature09041. PubMed PMID: 20505669; PubMed Central PMCID: PMCPMC4137042.
43. Terauchi A, Timmons KM, Kikuma K, Pechmann Y, Kneussel M, Umemori H. Selective synaptic targeting of the excitatory and inhibitory presynaptic organizers FGF22 and FGF7. J Cell Sci. 2015;128(2):281-92. Epub 2014/11/29. doi: 10.1242/jcs.158337. PubMed PMID: 25431136; PubMed Central PMCID: PMCPMC4294774.
44. Diniz LP, Almeida JC, Tortelli V, Vargas Lopes C, Setti-Perdigão P, Stipursky J, et al. Astrocyte-induced synaptogenesis is mediated by transforming growth factor β signaling through modulation of D-serine levels in cerebral cortex neurons. J Biol Chem. 2012;287(49):41432-45. Epub 2012/10/12. doi: 10.1074/jbc.M112.380824. PubMed PMID: 23055518; PubMed Central PMCID: PMCPMC3510841.
45. Fuentes-Medel Y, Ashley J, Barria R, Maloney R, Freeman M, Budnik V. Integration of a retrograde signal during synapse formation by glia-secreted TGF-β ligand. Curr Biol. 2012;22(19):1831-8. Epub 2012/09/11. doi: 10.1016/j.cub.2012.07.063. PubMed PMID: 22959350; PubMed Central PMCID: PMCPMC3605899.
46. Marqués G, Bao H, Haerry TE, Shimell MJ, Duchek P, Zhang B, et al. The Drosophila BMP type II receptor Wishful Thinking regulates neuromuscular synapse morphology and function. Neuron. 2002;33(4):529-43. Epub 2002/02/22. doi: 10.1016/s0896-6273(02)00595-0. PubMed PMID: 11856528.
47. Aberle H, Haghighi AP, Fetter RD, McCabe BD, Magalhães TR, Goodman CS. wishful thinking encodes a BMP type II receptor that regulates synaptic growth in Drosophila. Neuron. 2002;33(4):545-58. Epub 2002/02/22. doi: 10.1016/s0896-6273(02)00589-5. PubMed PMID: 11856529.
48. McCabe BD, Marqués G, Haghighi AP, Fetter RD, Crotty ML, Haerry TE, et al. The BMP homolog Gbb provides a retrograde signal that regulates synaptic growth at the Drosophila neuromuscular junction. Neuron. 2003;39(2):241-54. Epub 2003/07/23. doi: 10.1016/s0896-6273(03)00426-4. PubMed PMID: 12873382.
49. Goold CP, Davis GW. The BMP ligand Gbb gates the expression of synaptic homeostasis independent of synaptic growth control. Neuron. 2007;56(1):109-23. Epub 2007/10/09. doi: 10.1016/j.neuron.2007.08.006. PubMed PMID: 17920019; PubMed Central PMCID: PMCPMC2699048.
50. Ball RW, Warren-Paquin M, Tsurudome K, Liao EH, Elazzouzi F, Cavanagh C, et al. Retrograde BMP signaling controls synaptic growth at the NMJ by regulating trio expression in motor neurons. Neuron. 2010;66(4):536-49. Epub 2010/06/01. doi: 10.1016/j.neuron.2010.04.011. PubMed PMID: 20510858.

51. Xiao L, Michalski N, Kronander E, Gjoni E, Genoud C, Knott G, et al. BMP signaling specifies the development of a large and fast CNS synapse. Nat Neurosci. 2013;16(7):856-64. Epub 2013/05/28. doi: 10.1038/nn.3414. PubMed PMID: 23708139.
52. Berke B, Wittnam J, McNeill E, Van Vactor DL, Keshishian H. Retrograde BMP signaling at the synapse: a permissive signal for synapse maturation and activity-dependent plasticity. J Neurosci. 2013;33(45):17937-50. Epub 2013/11/08. doi: 10.1523/jneurosci.6075-11.2013. PubMed PMID: 24198381; PubMed Central PMCID: PMCPMC3818560.
53. Piccioli ZD, Littleton JT. Retrograde BMP signaling modulates rapid activity-dependent synaptic growth via presynaptic LIM kinase regulation of cofilin. J Neurosci. 2014;34(12):4371-81. Epub 2014/03/22. doi: 10.1523/jneurosci.4943-13.2014. PubMed PMID: 24647957; PubMed Central PMCID: PMCPMC3960475.
54. Ball RW, Peled ES, Guerrero G, Isacoff EY. BMP signaling and microtubule organization regulate synaptic strength. Neuroscience. 2015;291:155-66. Epub 2015/02/15. doi: 10.1016/j.neuroscience.2015.01.069. PubMed PMID: 25681521; PubMed Central PMCID: PMCPMC4369397.
55. Sulkowski MJ, Han TH, Ott C, Wang Q, Verheyen EM, Lippincott-Schwartz J, et al. A Novel, Noncanonical BMP Pathway Modulates Synapse Maturation at the Drosophila Neuromuscular Junction. PLoS Genet. 2016;12(1):e1005810. Epub 2016/01/28. doi: 10.1371/journal.pgen.1005810. PubMed PMID: 26815659; PubMed Central PMCID: PMCPMC4729469.
56. Higashi T, Tanaka S, Iida T, Okabe S. Synapse Elimination Triggered by BMP4 Exocytosis and Presynaptic BMP Receptor Activation. Cell Rep. 2018;22(4):919-29. Epub 2018/02/02. doi: 10.1016/j.celrep.2017.12.101. PubMed PMID: 29386134.
57. Kronander E, Clark C, Schneggenburger R. Role of BMP Signaling for the Formation of Auditory Brainstem Nuclei and Large Auditory Relay Synapses. Dev Neurobiol. 2019;79(2):155-74. Epub 2018/12/15. doi: 10.1002/dneu.22661. PubMed PMID: 30548566.
58. Kim N, Kim S, Nahm M, Kopke D, Kim J, Cho E, et al. BMP-dependent synaptic development requires Abi-Abl-Rac signaling of BMP receptor macropinocytosis. Nat Commun. 2019;10(1):684. Epub 2019/02/10. doi: 10.1038/s41467-019-08533-2. PubMed PMID: 30737382; PubMed Central PMCID: PMCPMC6368546.
59. Vicidomini R, Serpe M. Local BMP signaling: A sensor for synaptic activity that balances synapse growth and function. Curr Top Dev Biol. 2022;150:211-54. Epub 2022/07/12. doi: 10.1016/bs.ctdb.2022.04.001. PubMed PMID: 35817503.
60. Zhang F, Endo S, Cleary LJ, Eskin A, Byrne JH. Role of transforming growth factor-beta in long-term synaptic facilitation in Aplysia. Science. 1997;275(5304):1318-20. Epub 1997/02/28. doi: 10.1126/science.275.5304.1318. PubMed PMID: 9036859.
61. Heupel K, Sargsyan V, Plomp JJ, Rickmann M, Varoqueaux F, Zhang W, et al. Loss of transforming growth factor-beta 2 leads to impairment of central synapse function. Neural Dev. 2008;3:25. Epub 2008/10/16. doi: 10.1186/1749-8104-3-25. PubMed PMID: 18854036; PubMed Central PMCID: PMCPMC2576228.
62. Fong SW, McLennan IS, McIntyre A, Reid J, Shennan KI, Bewick GS. TGF-beta2 alters the characteristics of the neuromuscular junction by regulating presynaptic quantal size. Proc Natl Acad Sci U S A. 2010;107(30):13515-9. Epub 2010/07/14. doi: 10.1073/pnas.1001695107. PubMed PMID: 20624974; PubMed Central PMCID: PMCPMC2922142.
63. Krieglstein K, Zheng F, Unsicker K, Alzheimer C. More than being protective: functional roles for TGF-

β/activin signaling pathways at central synapses. Trends Neurosci. 2011;34(8):421-9. Epub 2011/07/12. doi: 10.1016/j.tins.2011.06.002. PubMed PMID: 21742388.
64. Araujo AP, Diniz LP, Eller CM, de Matos BG, Martinez R, Gomes FC. Effects of Transforming Growth Factor Beta 1 in Cerebellar Development: Role in Synapse Formation. Front Cell Neurosci. 2016;10:104. Epub 2016/05/21. doi: 10.3389/fncel.2016.00104. PubMed PMID: 27199658; PubMed Central PMCID: PMCPMC4846658.
65. Luo SX, Timbang L, Kim JI, Shang Y, Sandoval K, Tang AA, et al. TGF-β Signaling in Dopaminergic Neurons Regulates Dendritic Growth, Excitatory-Inhibitory Synaptic Balance, and Reversal Learning. Cell Rep. 2016;17(12):3233-45. Epub 2016/12/24. doi: 10.1016/j.celrep.2016.11.068. PubMed PMID: 28009292; PubMed Central PMCID: PMCPMC5312261.
66. Gottmann K, Mittmann T, Lessmann V. BDNF signaling in the formation, maturation and plasticity of glutamatergic and GABAergic synapses. Exp Brain Res. 2009;199(3-4):203-34. Epub 2009/09/25. doi: 10.1007/s00221-009-1994-z. PubMed PMID: 19777221.
67. Pozzo-Miller LD, Gottschalk W, Zhang L, McDermott K, Du J, Gopalakrishnan R, et al. Impairments in high-frequency transmission, synaptic vesicle docking, and synaptic protein distribution in the hippocampus of BDNF knockout mice. J Neurosci. 1999;19(12):4972-83. Epub 1999/06/15. doi: 10.1523/jneurosci.19-12-04972.1999. PubMed PMID: 10366630; PubMed Central PMCID: PMCPMC6782660.
68. Luikart BW, Nef S, Virmani T, Lush ME, Liu Y, Kavalali ET, et al. TrkB has a cell-autonomous role in the establishment of hippocampal Schaffer collateral synapses. J Neurosci. 2005;25(15):3774-86. Epub 2005/04/15. doi: 10.1523/jneurosci.0041-05.2005. PubMed PMID: 15829629; PubMed Central PMCID: PMCPMC6724922.
69. Parkhurst CN, Yang G, Ninan I, Savas JN, Yates JR, 3rd, Lafaille JJ, et al. Microglia promote learning-dependent synapse formation through brain-derived neurotrophic factor. Cell. 2013;155(7):1596-609. Epub 2013/12/24. doi: 10.1016/j.cell.2013.11.030. PubMed PMID: 24360280; PubMed Central PMCID: PMCPMC4033691.
70. Davis-López de Carrizosa MA, Morado-Díaz CJ, Morcuende S, de la Cruz RR, Pastor AM. Nerve growth factor regulates the firing patterns and synaptic composition of motoneurons. J Neurosci. 2010;30(24):8308-19. Epub 2010/06/18. doi: 10.1523/jneurosci.0719-10.2010. PubMed PMID: 20554882; PubMed Central PMCID: PMCPMC6634574.
71. Newbern JM, Li X, Snider WD. Signaling endosomes trigger synapse assembly. Neuron. 2010;67(3):352-4. Epub 2010/08/11. doi: 10.1016/j.neuron.2010.07.025. PubMed PMID: 20696371.
72. Grantyn R, Henneberger C, Jüttner R, Meier JC, Kirischuk S. Functional hallmarks of GABAergic synapse maturation and the diverse roles of neurotrophins. Front Cell Neurosci. 2011;5:13. Epub 2011/07/21. doi: 10.3389/fncel.2011.00013. PubMed PMID: 21772813; PubMed Central PMCID: PMCPMC3131524.
73. Sharma N, Deppmann CD, Harrington AW, St Hillaire C, Chen ZY, Lee FS, et al. Long-distance control of synapse assembly by target-derived NGF. Neuron. 2010;67(3):422-34. doi: 10.1016/j.neuron.2010.07.018. PubMed PMID: 20696380; PubMed Central PMCID: PMC2949359.
74. Gibon J, Barker PA. Neurotrophins and Proneurotrophins: Focus on Synaptic Activity and Plasticity in the Brain. Neuroscientist. 2017;23(6):587-604. Epub 2017/03/18. doi: 10.1177/1073858417697037. PubMed PMID: 28303740.
75. Lehigh KM, West KM, Ginty DD. Retrogradely Transported TrkA Endosomes Signal Locally within Dendrites to Maintain Sympathetic Neuron Synapses. Cell Rep. 2017;19(1):86-100. Epub 2017/04/06.

doi: 10.1016/j.celrep.2017.03.028. PubMed PMID: 28380365; PubMed Central PMCID: PMCPMC5562950.
76. Ledda F, Paratcha G, Sandoval-Guzmán T, Ibáñez CF. GDNF and GFRalpha1 promote formation of neuronal synapses by ligand-induced cell adhesion. Nat Neurosci. 2007;10(3):293-300. Epub 2007/02/21. doi: 10.1038/nn1855. PubMed PMID: 17310246.
77. Bonafina A, Trinchero MF, Ríos AS, Bekinschtein P, Schinder AF, Paratcha G, et al. GDNF and GFRα1 Are Required for Proper Integration of Adult-Born Hippocampal Neurons. Cell Rep. 2019;29(13):4308-19.e4. Epub 2019/12/26. doi: 10.1016/j.celrep.2019.11.100. PubMed PMID: 31875542.
78. Stanga S, Boido M, Kienlen-Campard P. How to Build and to Protect the Neuromuscular Junction: The Role of the Glial Cell Line-Derived Neurotrophic Factor. Int J Mol Sci. 2020;22(1). Epub 2020/12/31. doi: 10.3390/ijms22010136. PubMed PMID: 33374485; PubMed Central PMCID: PMCPMC7794999.
79. Fernández-Suárez D, Krapacher FA, Pietrajtis K, Andersson A, Kisiswa L, Carrier-Ruiz A, et al. Adult medial habenula neurons require GDNF receptor GFRα1 for synaptic stability and function. PLoS Biol. 2021;19(11):e3001350. Epub 2021/11/09. doi: 10.1371/journal.pbio.3001350. PubMed PMID: 34748545; PubMed Central PMCID: PMCPMC8601618.
80. Wilson SI, Shafer B, Lee KJ, Dodd J. A molecular program for contralateral trajectory: Rig-1 control by LIM homeodomain transcription factors. Neuron. 2008;59(3):413-24. Epub 2008/08/15. doi: 10.1016/j.neuron.2008.07.020. PubMed PMID: 18701067.
81. Wu Z, Makihara S, Yam PT, Teo S, Renier N, Balekoglu N, et al. Long-Range Guidance of Spinal Commissural Axons by Netrin1 and Sonic Hedgehog from Midline Floor Plate Cells. Neuron. 2019;101(4):635-47.e4. Epub 2019/01/22. doi: 10.1016/j.neuron.2018.12.025. PubMed PMID: 30661738.
82. Wei P, Smeyne RJ, Bao D, Parris J, Morgan JI. Mapping of Cbln1-like immunoreactivity in adult and developing mouse brain and its localization to the endolysosomal compartment of neurons. Eur J Neurosci. 2007;26(10):2962-78. Epub 2007/11/16. doi: 10.1111/j.1460-9568.2007.05913.x. PubMed PMID: 18001291.
83. Ibata K, Kono M, Narumi S, Motohashi J, Kakegawa W, Kohda K, et al. Activity-Dependent Secretion of Synaptic Organizer Cbln1 from Lysosomes in Granule Cell Axons. Neuron. 2019;102(6):1184-98.e10. Epub 2019/05/11. doi: 10.1016/j.neuron.2019.03.044. PubMed PMID: 31072786.
84. Shao Q, Yang M, Liang C, Ma L, Zhang W, Jiang Z, et al. C9orf72 and smcr8 mutant mice reveal MTORC1 activation due to impaired lysosomal degradation and exocytosis. Autophagy. 2020;16(9):1635-50. Epub 2019/12/19. doi: 10.1080/15548627.2019.1703353. PubMed PMID: 31847700; PubMed Central PMCID: PMCPMC8386604.
85. Williams SE, Mann F, Erskine L, Sakurai T, Wei S, Rossi DJ, et al. Ephrin-B2 and EphB1 mediate retinal axon divergence at the optic chiasm. Neuron. 2003;39(6):919-35. Epub 2003/09/16. doi: 10.1016/j.neuron.2003.08.017. PubMed PMID: 12971893.
86. Peng J, Fabre PJ, Dolique T, Swikert SM, Kermasson L, Shimogori T, et al. Sonic Hedgehog Is a Remotely Produced Cue that Controls Axon Guidance Trans-axonally at a Midline Choice Point. Neuron. 2018;97(2):326-40 e4. Epub 2018/01/19. doi: 10.1016/j.neuron.2017.12.028. PubMed PMID: 29346753.
87. Lustig M, Erskine L, Mason CA, Grumet M, Sakurai T. Nr-CAM expression in the developing mouse nervous system: ventral midline structures, specific fiber tracts, and neuropilar regions. J Comp

Neurol. 2001;434(1):13-28. Epub 2001/05/01. doi: 10.1002/cne.1161. PubMed PMID: 11329126.
88. Marcucci F, Soares CA, Mason C. Distinct timing of neurogenesis of ipsilateral and contralateral retinal ganglion cells. J Comp Neurol. 2019;527(1):212-24. Epub 2018/05/16. doi: 10.1002/cne.24467. PubMed PMID: 29761490; PubMed Central PMCID: PMCPMC6237670.
89. Haddick PC, Tom I, Luis E, Quiñones G, Wranik BJ, Ramani SR, et al. Defining the ligand specificity of the deleted in colorectal cancer (DCC) receptor. PLoS One. 2014;9(1):e84823. Epub 2014/01/09. doi: 10.1371/journal.pone.0084823. PubMed PMID: 24400119; PubMed Central PMCID: PMCPMC3882260 following conflicts: All of the authors are or have been employed by Genentech/Roche and may own shares in the company. All funding for this research was provided by Genentech/Roche. This does not alter the authors' adherence to all the PLOS ONE policies on sharing data and materials.
90. Bao D, Pang Z, Morgan MA, Parris J, Rong Y, Li L, et al. Cbln1 is essential for interaction-dependent secretion of Cbln3. Mol Cell Biol. 2006;26(24):9327-37. doi: 10.1128/MCB.01161-06. PubMed PMID: 17030622; PubMed Central PMCID: PMC1698530.
91. Rong Y, Wei P, Parris J, Guo H, Pattarini R, Correia K, et al. Comparison of Cbln1 and Cbln2 functions using transgenic and knockout mice. J Neurochem. 2012;120(4):528-40. Epub 2011/11/29. doi: 10.1111/j.1471-4159.2011.07604.x. PubMed PMID: 22117778; PubMed Central PMCID: PMCPMC3259274.
92. Yang M, Cagle MC, Honig MG. Identification of cerebellin2 in chick and its preferential expression by subsets of developing sensory neurons and their targets in the dorsal horn. J Comp Neurol. 2010;518(14):2818-40. doi: 10.1002/cne.22366. PubMed PMID: 20506477; PubMed Central PMCID: PMC2880495.
